# Skin toxicity induced by chemotherapy or molecular targeted therapy combined with immune checkpoint inhibitors in Asian patients: A literature review by the Japanese Pharmacist-led Oncodermatology Study Team

**DOI:** 10.1007/s10147-026-03044-9

**Published:** 2026-04-16

**Authors:** Yohei Iimura, Junichi Higuchi, Akimitsu Maeda, Kazuhiro Shimomura, Hirotoshi Iihara, Hironori Fujii, Takuya Iwamoto, Yoshitaka Saito, Hisanaga Nomura, Keiko Komori, Hidenori Tokuda, Ryuta Urakawa, Tatsuya Sumiya, Ryosuke Yanai, Mariko Kono, Masaki Ihira, Tomohiro Kurokawa, Yuya Ishii, Masanobu Uchiyama, Teppei Yamada, Yasumasa Tsuda, Yusuke Tsuchiya, Toshinobu Hayashi, Seiichiro Kuroda

**Affiliations:** 1 Department of Pharmacy, The IMSUT Hospital, The Institute of Medical Science, The University of Tokyo, 4-6-1, Shirokanedai, Minato-Ku, Tokyo , 108-8639 Japan; 2 Department of Pharmacy, Ohara Healthcare Foundation Kurashiki Central Hospital, Kurashiki, Japan; 3 Department of Pharmacy, Aichi Cancer Center Hospital, Nagoya, Japan; 4 Department of Pharmacy, Gifu University Hospital, Gifu-shi, Japan; 5 Department of Pharmacy, Mie University Hospital, Tsu, Japan; 6 Department of Clinical Pharmaceutics & Therapeutics, Faculty of Pharmaceutical Sciences, Hokkaido University of Science, Sapporo, Japan; 7 Department of Clinical Pharmacology and Therapeutics, Kyoto University Hospital, Kyoto, Japan; 8 Department of Pharmacy, Japanese Red Cross Kyoto Daiichi Hospital, Kyoto, Japan; 9 Department of Pharmacy, The University of Osaka Dental Hospital, Suita, Japan; 10 Department of Clinical Pharmacy Research and Education, Graduate School of Pharmaceutical Sciences, The University of Osaka, Suita, Japan; 11 Department of Pharmacy, Yokohama City Minato Red Cross Hospital, Yokohama, Japan; 12 Department of Pharmacy, Tokyo Metropolitan Police Hospital, Nakano, Japan; 13 Department of Surgery, Jyoban Hospital of Tokiwa Foundation, Iwaki, Japan; 14 Department of Pharmacy, Jyoban Hospital of Tokiwa Foundation, Iwaki, Japan; 15 Department of Oncology and Infectious Disease Pharmacy, Faculty of Pharmaceutical Sciences, Fukuoka University, Fukuoka, Japan; 16 Department of Gastroenterological Surgery, Faculty of Medicine, Fukuoka University, Fukuoka, Japan; 17 Department of Pharmacy, St. Luke’s International Hospital, Tokyo, Japan; 18 Department of Pharmacy, Ageo Central General Hospital, Ageo, Japan; 19 Department of Pharmaceutical Sciences for Health Crisis Management, Faculty of Pharmaceutical Sciences, Fukuoka University, Fukuoka, Japan

**Keywords:** Skin toxicity, Chemotherapy, Molecular targeted therapy, Immune checkpoint inhibitors, Literature review

## Abstract

**Background:**

Combination therapy with immune checkpoint inhibitors (ICIs) and cytotoxic or targeted anticancer agents has improved survival in multiple cancers but may exacerbate drug-related skin toxicities. These toxicities share inflammatory mechanisms, and ICI-induced immune cell infiltration into the skin may increase both their incidence and severity. This review evaluated the impact of adding ICIs on skin toxicities in adult Asian patients**.**

**Methods:**

PubMed was searched for English-language clinical trials published between January 2014 and September 2025 using the keywords “immune checkpoint inhibitor” and “clinical study.” Randomized controlled trials involving adult Asian patients treated with ICIs in combination with cytotoxic chemotherapy or molecularly targeted therapy were included.

**Results:**

Of 7287 identified articles, 28 met inclusion criteria. Studies assessed capecitabine-induced hand–foot syndrome (HFS), multikinase inhibitor–induced hand–foot skin reaction (HFSR), taxane-induced alopecia, and epidermal growth factor receptor (EGFR) inhibitor-related skin toxicities. The incidence of capecitabine-related HFS (11/13 articles) and EGFR inhibitor-related skin toxicities (1/1 article) tended to be higher with the addition of ICIs.

**Conclusions:**

While ICIs have substantially improved survival outcomes, their immunomodulatory effects may amplify drug-specific dermatologic toxicities when used in combination regimens. Shared inflammatory pathways and immune cell recruitment to the skin likely underlie this interaction, underscoring the importance of anticipatory monitoring and optimized management strategies in combination therapy.

## Introduction

Immune checkpoint inhibitors (ICIs) have revolutionized cancer therapy by improving survival outcomes across multiple tumor types. In the ATTRACTION-4 trial, adding nivolumab to standard chemotherapy significantly improved progression-free survival (PFS) in patients with HER2-negative, previously untreated, unresectable, advanced, or recurrent gastric or gastroesophageal junction cancer [1]. Building on the established efficacy of cytotoxic chemotherapy (CTx) and molecularly targeted therapy (MTT), numerous clinical trials have demonstrated that combining ICIs with chemotherapy often yields synergistic therapeutic benefits. However, these regimens may also increase the risk of severe, sometimes unmanageable toxicities, potentially necessitating treatment discontinuation. Clinical trials in Asian patients have reported an increase in skin toxicities when ICIs are added to chemotherapy [2]. In particular, a subgroup analysis of Asian patients indicated a higher incidence of hand-foot syndrome (HFS) with ICI combination therapy [3, 4]. These findings suggest that in Asian patients, combining chemotherapy with ICIs may exacerbate chemotherapy-induced skin toxicity. Similar effects are suspected with molecularly targeted drugs, necessitating further investigation.

Skin toxicity is among the most clinically significant adverse events induced by ICIs. Evidence suggests that adding or sequentially administering ICIs may increase both the incidence and severity of skin adverse events compared with monotherapy. For instance, in patients with unresectable advanced or recurrent gastric or gastroesophageal junction cancer, adding nivolumab to chemotherapy improved PFS but was associated with increased skin toxicities [2]. Similarly, administering enfortumab vedotin (EV) in combination with, or following, ICIs may increase skin toxicity compared with EV monotherapy [5]. In advanced non-small cell lung cancer, adding ICIs to chemotherapy, with or without antiangiogenic therapy, increases the incidence of rash [6]. Dual ICI regimens, such as nivolumab plus ipilimumab, are also associated with higher rates of rash [7], and concomitant chemotherapy further amplifies the risk [8]. These effects are attributed both to the intrinsic adverse effects of ICIs (e.g., rash and pruritus) and to ICI-induced alterations in the cutaneous immune environment [9]. Although the exacerbation of skin toxicity with combination or sequential ICI therapy is clinically recognized, its overall burden remains poorly defined. Limited recommendations have been reported for the management of skin toxicities associated with the combination of EV and ICIs [10], and to our knowledge, no recommendation addresses skin toxicities arising from sequential chemotherapy administered following ICI treatment. Consequently, no standardized management strategies have been established. Literature indicates that Asian patients tend to experience a relatively higher incidence of HFS, hand-foot skin reaction (HFSR), and EGFR inhibitor-related skin toxicities compared with other populations. According to the literature review we reported, patients in Asia tend to exhibit a relatively higher incidence of HFS, HFSR, and epidermal growth factor receptor (EGFR) inhibitor-related skin toxicity compared to other ethnicities, highlighting the need for management approaches tailored to this group [11, 12]. To address this gap, we conducted a literature review to characterize the landscape of skin toxicity induced by chemotherapy and molecular targeted therapy combined with ICIs for patients in Asia. Asian skin is characterized by a relatively thin stratum corneum and densely packed keratinocytes, which confer increased sensitivity and a heightened reactivity to external stimuli [13, 14]. At the same time, higher melanin levels in Asian skin provide some natural protection against ultraviolet radiation [15]. Conversely, individuals of Western descent, who generally have lower melanin content, are more susceptible to hyperpigmentation, including that induced by 5-fluorouracil and corticosteroid therapies. These differences underscore the importance of evaluating skin toxicity in a race- and skin type-specific context. Accordingly, this literature review focuses specifically on studies involving Asian populations.

## Methods

### Information sources and search strategy

The literature search, conducted in PubMed, covered the period from January 2014 to September 2025 and included only articles published in English. The search strategy focused on clinical trials, using the following keywords: “immune checkpoint inhibitor AND clinical study.” Two authors (YI and JH) independently screened titles and abstracts, followed by a full-text review according to predefined inclusion and exclusion criteria. In addition, both authors were allowed to conduct manual searches to identify additional eligible articles. Reasons for exclusion were documented, and any discrepancies were resolved through consultation with a third author.

### Selection criteria

The inclusion criteria were as follows: (i) employing randomized controlled trial (RCT); and (ii) involving adult patients who received CTx or MTT in combination with ICIs. (iii) focusing exclusively on RCTs that included Asian populations. Subgroup analyses of Asian populations within RCTs were permitted as valid for analysis. The exclusion criteria were as follows: (i) study protocols; (ii) meta-analyses; (iii) guidelines and reviews; (iv) letters; (v) case reports; (vi) basic science studies; (vii) studies combining ICIs with radiation therapy; (viii) studies evaluating ICIs alone; (ix) trials of combinations in which the additional agents do not cause skin disorders when used alone; and (x) trials in which the control group received a different anticancer agent for CICs.

## Results

### Literature‑search results

**Fig. 1:**
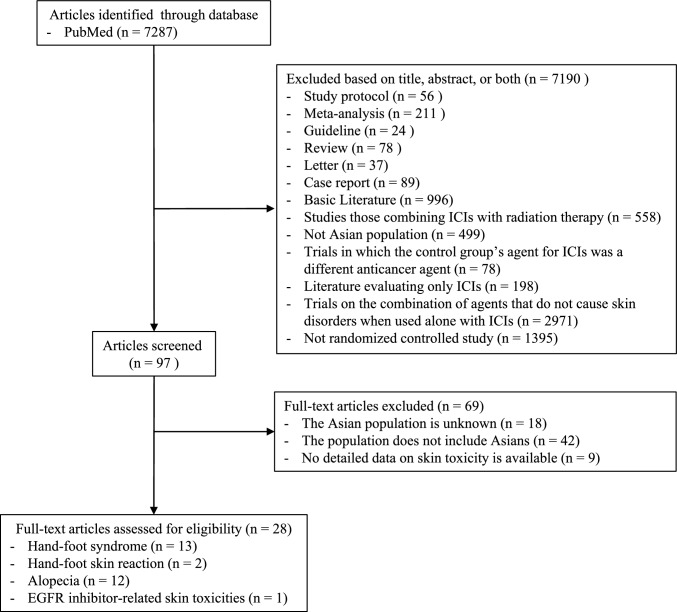
Diagram of study selection/screening process

In total, 7287 articles published between January 2014 and September 2025 were identified through PubMed. Following title and abstract screening, 7190 publications were excluded due to irrelevance to the research topic. An additional 69 articles were excluded after full-text review because they lacked usable data. Ultimately, 12 articles on HFS, 2 on HFSR, 9 on taxane-induced alopecia, and 1 on EGFR inhibitor-related skin toxicity were included. The literature screening process is summarized in Fig. 1.

### Incidence of skin toxicities

The review focused on studies reporting HFS induced by capecitabine, HFSR induced by multi-kinase inhibitors (MKIs), alopecia induced by taxanes, and EGFR inhibitor-related skin toxicities. No studies reporting skin toxicities associated with EV were identified.

### HFS induced by capecitabine

A higher incidence of HFS tended to be reported in several of the included studies. Among patients receiving capecitabine in combination with ICIs, HFS occurred in 12–40%, compared with 11–49% in patients not receiving ICIs. The incidence of grade ≥ 3 HFS was 0–5.6% with concomitant ICI use and 0–3.3% in patients without concomitant ICI treatment (Table 1).

**Table 1:** Summary of the incidence of capecitabine-induced HFS when combined with ICIs

Reference number	Year	Combined ICIs	Number of patients	Proportion of Asian patients	Study type	Incidence of HFS (%)			
						Any grade		≥ Grade 3	
						With ICIs	Without ICIs	With ICIs	Without ICIs
[51]	2025	Pembrolizumab	143	48	RCT	16	14	< 1	< 1
[52]	2025	Pembrolizumab	101	100	RCT (Japanese subgroup analysis)	37.5	49.1	NA	NA
[1]	2024	Nivolumab	362	100	RCT	13.6	13.1	1.1	1.7
[53]	2024	Nivolumab	912	100	RCT	14	12	0	0
[3]	2023	Pembrolizumab	1579	34	RCT	21	19	2	3
[54]	2023	Pembrolizumab	119	100	RCT (Asian subgroup analysis)	40.3	36.8	NA	NA
[55]	2022	Nivolumab	205	100	RCT (Chinese subgroup analysis)	12	14	2	1
[56]	2022	Nivolumab	724	100	RCT	13	12	1	1
[57]	2020	Pembrolizumab	507	24.7	RCT	23.6	18	4.8	3.3
[58]	2024	Nivolumab	1549	24	RCT	13	11	2	1
[4]	2025	Pembrolizumab	236	100	RCT (Chinese subgroup analysis)	25.6	17.3	5.6	0.9
[59]	2023	Pembrolizumab	696	34	RCT	22	21	1	1
[60]	2024	Tislelizumab	992	75	RCT	19.1	18.8	3	2

HFS, hand–foot syndrome; ICIs, immune check point inhibitors

### HFSR induced by MKIs

One of the two included studies reported a numerically higher incidence of HFSR in patients receiving MKIs combined with ICIs. Among patients receiving MKIs in combination with ICIs, the incidence of HFSR was 28–39%, compared with 27–41% in patients not receiving ICIs (Table 2).

**Table 2:** Summary of the incidence of MKI-induced HFSR when combined with ICIs

Reference number	Year	Agent	Combined ICIs	Number of patients	Proportion of Asian patients	Study type	Incidence of HFSR (%)			
							Any grade		> Grade 3	
							With ICIs	Without ICIs	With ICIs	Without ICIs
[61]	2023	Lenvatinib	Pembrolizumab	790	43.5	RCT	28	27	5	3
[62]	2023	Cabozantinib	Atezolizumab	518	10.7	RCT	39	41	NA	NA

MKIs, multi-kinase inhibitors; HFSR, hand–foot skin reaction; ICIs, immune check point inhibitors

### Alopecia induced by taxane

Five of the 12 included studies reported a numerically higher incidence of alopecia in patients receiving taxanes combined with ICIs. The incidence of alopecia ranged from 9 to 94% in patients receiving taxanes in combination with ICIs, compared with 8–95% in those without concomitant ICI exposure. The incidence of grade ≥ 3 alopecia was 0–0.4% in patients with concomitant ICI use and 0–0.6% in patients without concomitant ICI treatment (Table 3).

**Table 3:** Summary of the incidence of taxane-induced alopecia when administered with ICIs

Reference number	Year	Taxane	Combined ICIs	Number of patients	Proportion of Asian patients	Study type	Incidence of alopecia (%)			
							Any grade		> Grade 3	
							With ICIs	Without ICIs	With ICIs	Without ICIs
[63]	2022	Paclitaxel	Pembrolizumab	57	100	RCT (Japanese subgroup analysis)	89	77	0	0
[64]	2022	nab-paclitaxel	Atezolizumab	36	100	RCT (Japanese subgroup analysis)	94	95	0	0
[65]	2021	nab-paclitaxel	Atezolizumab	902	17.8	RCT	57.2	57.4	0.4	0.2
[66]	2025	Docetaxel	Pembrolizumab	1028	15	RCT	34.6	36.6	0.2	0.6
[67]	2019	nab-paclitaxel	Atezolizumab	890	17.8	RCT	57	57	< 1	< 1
[68]	2020	nab-paclitaxel, paclitaxel	Pembrolizumab	843	20.7	RCT	33	33	1	1
[69]	2019	nab-paclitaxel	Atezolizumab	705	2.2	RCT	31	26	0	0
[70]	2017	Paclitaxel	Ipilimumab	749	28.6	RCT	9	8	0	0
[71]	2021	Paclitaxel	Nivolumab	548	100	RCT	52.4	54.5	0	0
[72]	2022	Paclitaxel	Toripalimab	514	100	RCT	35.4	40.5	0	0
[73]	2021	Paclitaxel	Tislelizumab	355	100	RCT	64.2	61.5	0	0
[73]	2021	nab-paclitaxel	Tislelizumab	355	100	RCT	69.5	61.5	0	0
[74]	2023	Paclitaxel	Tislelizumab	645	75	RCT	18	20	0	0

ICIs, immune check point inhibitors

### EGFR inhibitor-induced skin toxicities

Only one study was identified. In this study, the incidence of rash was 67% in patients receiving EGFR inhibitor (osimertinib) in combination with ICIs, compared with 53% in patients not receiving concomitant ICIs. The incidence of grade ≥ 3 rash was 0% both in patients receiving ICIs in combination with osimertinib and in those without concomitant ICI treatment. The incidence of pruritus was 42% in patients receiving ICIs in combination with EGFR inhibitors and 24% in patients without concomitant ICI exposure. The incidence of grade ≥ 3 pruritus was 0% both in patients receiving ICIs in combination with osimertinib and in those without concomitant ICI treatment (Table 4).

**Table 4:** Summary of the incidence of EGFR inhibitor (osimertinib)-induced skin toxicities when administered with ICIs

Reference number	Year	Combined ICIs	Number of patients	Proportion of Asian patients	Study type	Incidence of skin toxicities (%)			
						Any grade		> Grade 3	
						With ICIs	Without ICIs	With ICIs	Without ICIs
[75]	2019	Durvalumab	29	89.7	RCT	Rash			
						67	53	0	0
						Pruritus			
						42	24	0	0
						Dry skin			
						33	18	0	0
						Paronychia			
						17	41	0	0

EGFR, epidermal growth factor receptor; ICIs, immune check point inhibitors

## Discussion

We conducted a literature review to investigate the incidence of skin toxicities induced by CTx or MTT combined with ICIs. The incidence of capecitabine-induced HFS tended to be higher when combined with ICIs. Similarly, EGFR inhibitor–related skin toxicities tended to be higher with concomitant ICI use. In contrast, the incidence of MKI-related HFSR and taxane-induced alopecia was not increased when combined with ICIs. These findings should be interpreted as descriptive observations, as much of the evidence was derived from subgroup or secondary analyses.

**Fig. 2:**
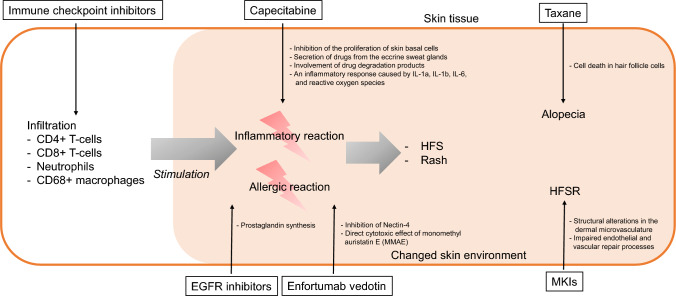
Conceptual overview of potential mechanisms of skin toxicity induced by chemotherapy or molecular-targeted therapy combined with immune checkpoint inhibitors (ICIs) Capecitabine induces reactive oxygen species and interleukin-mediated inflammatory responses, leading to hand–foot syndrome (HFS). Epidermal growth factor receptor (EGFR) inhibitors cause cutaneous toxicity through EGF signaling inhibition and prostaglandin-mediated inflammation. Enfortumab vedotin (EV) induces skin toxicity via Nectin-4 inhibition, with its payload, monomethyl auristatin E (MMAE), contributing direct cytotoxic effects. Multi-kinase inhibitor (MKI)-induced hand–foot skin reaction (HFSR) arises from structural changes in dermal microvasculature and impaired vascular repair, while taxane-related alopecia results from apoptosis of hair follicle cells. These mechanisms are conceptual and based on observations discussed in the manuscript, suggesting that ICI-mediated immune modulation may trigger or exacerbate skin toxicities associated with inflammatory pathways. In contrast, inflammatory responses are not implicated in HFSR or alopecia

These differences may be partly explained by immune modulation induced by ICIs. ICI therapy enhances cutaneous immune activity, including infiltration of CD4⁺ and CD8⁺ T cells and other inflammatory cells [9, 16, 17]. Such immune activation may predispose the skin to inflammatory and hypersensitivity reactions, potentially exacerbating dermatologic toxicities driven by inflammatory mechanisms. Similar mechanisms may operate when ICIs are combined with CTx or MTT that induce skin toxicity through inflammatory pathways. Previous immunohistological studies have demonstrated various patterns of immune cell infiltration in ICI-related cutaneous toxicities, including CD3⁺/CD4⁺ predominant infiltration and mixed CD4⁺/CD8⁺ populations [17–22]. These toxicities often occur early after ICI initiation [19, 23–28]. Even in the absence of overt ICI-related eruptions, prior ICI exposure may modify the immune milieu and increase susceptibility to inflammatory skin toxicities induced by concomitant therapies (Fig. 2). Although the precise mechanisms underlying EGFR inhibitor–associated skin toxicities remain incompletely understood, inflammatory responses mediated by prostaglandin synthesis and immune modulation have been implicated [29–33]. Similarly, several mechanisms have been proposed for HFS, including suppression of basal keratinocyte proliferation, drug excretion through eccrine sweat glands, local toxicity from drug metabolites, and inflammatory responses mediated by cytokines and reactive oxygen species [34–38]. Preventive strategies targeting inflammatory pathways, such as topical diclofenac or hydrocortisone, have shown efficacy in reducing the incidence of HFS [39, 40], and oral nonsteroidal anti-inflammatory drugs may also be beneficial in preventing EGFR inhibitor–related skin toxicities [41, 42].

In contrast, HFSR associated with MKIs is thought to result from inhibition of multiple receptor tyrosine kinases, leading to structural alterations in the dermal microvasculature and impaired endothelial and vascular repair processes [43, 44]. This mechanism may explain why the incidence of MKI-related HFSR did not increase with the addition of ICIs in the studies reviewed. Similarly, taxane-induced alopecia results from disruption of microtubule assembly, leading to apoptosis of hair follicle cells that remain predominantly in the anagen phase [45, 46]. Although data on enfortumab vedotin (EV)-related skin toxicities were not included in the current review, previous studies—including our own work [5]—suggest that EV-associated skin toxicity may result from Nectin-4 inhibition. Because Nectin-4 is expressed in normal epidermal and adnexal tissues [47, 48], EV may exert cytotoxic effects on normal skin, potentially triggering inflammatory and hypersensitivity reactions [49, 50]. This discussion is intended to provide context and hypothesis-generating insights rather than definitive conclusions.

Ethnic differences in skin structure may also influence susceptibility to dermatologic toxicities. Asians have a thinner stratum corneum and higher melanin content, which could hypothetically predispose them to more pronounced superficial inflammatory reactions and accentuated color changes. These considerations are speculative and intended to generate hypotheses rather than definitive conclusions. However, the available evidence remains limited, and additional studies, including real-world data, are needed to better characterize these associations.

## Management recommendations

For HFS and EGFR inhibitor–related skin toxicities, intensive prevention measures and patient self-care education should be emphasized, with prompt intervention at onset, as ICI administration may be associated with a tendency toward higher frequency and severity.

## Limitations

This literature review has some limitations. Formal quality assessment tools, such as GRADE or the Cochrane risk-of-bias tool, were not employed. Future efforts should aim to establish evidence-based guidelines for Asian populations through systematic reviews conducted under more rigorous methodological frameworks. In addition, further investigations that account for the distinct skin characteristics across global populations are required to develop treatment strategies that are both regionally tailored and globally applicable. Addressing these research gaps may ultimately lead to better patient outcomes and more effective management of treatment-related skin toxicities. ICIs may exacerbate skin toxicities when administered concurrently with other anticancer therapies. Limited data also suggest that sequential administration of anticancer agents following ICIs could further increase the risk of skin toxicity. Although Tables 1–4 compare the frequencies of skin toxicities between patients receiving systemic therapy with or without ICIs, the small sample sizes in the included studies preclude reliable calculation of odds ratios or confidence intervals, and a formal meta-analytic approach was not feasible. Therefore, the findings should be interpreted as descriptive observations. Consequently, systematic investigation of skin toxicities associated with CTx or MTT administered after ICIs is warranted.

## Data Availability

The data supporting the findings of this study are available from the corresponding author, YI, upon reasonable request.

## References

[1] Boku N, Omori T, Shitara K et al (2024) Nivolumab plus chemotherapy in patients with HER2-negative, previously untreated, unresectable, advanced, or recurrent gastric/gastroesophageal junction cancer: 3-year follow-up of the ATTRACTION-4 randomized, double-blind, placebo-controlled, phase 3 trial. Gastric Cancer 27:1287–1301. 10.1007/s10120-024-01535-0 39162872 10.1007/s10120-024-01535-0 PMC11513732

[2] Kang YK, Ryu MH, Oh DY et al (2025) Nivolumab plus chemotherapy versus placebo plus chemotherapy in Korean patients with HER2-negative, untreated, unresectable advanced or recurrent gastric or gastroesophageal junction cancer: subgroup analysis of a randomized, multicenter, double-blind phase 3 trial (ATTRACTION-4). Cancer Res Treat. 10.4143/crt.2024.913 40610007 10.4143/crt.2024.913 PMC13382558

[3] Rha SY, Oh DY, Yañez P et al (2023) Pembrolizumab plus chemotherapy versus placebo plus chemotherapy for HER2-negative advanced gastric cancer (KEYNOTE-859): a multicentre, randomised, double-blind, phase 3 trial. Lancet Oncol 24:1181–1195. 10.1016/S1470-2045(23)00515-6 37875143 10.1016/S1470-2045(23)00515-6

[4] Qin S, Bai Y, Li J et al (2025) First-line Pembrolizumab plus chemotherapy for HER2-negative advanced gastric cancer: China subgroup analysis of the randomized phase 3 KEYNOTE-859 study. Adv Ther 42:1892–1906. 10.1007/s12325-024-03069-4 40025394 10.1007/s12325-024-03069-4 PMC11929688

[5] Iimura Y, Kuroda S, Kaichi S et al (2025) A literature review and management approach for severe skin toxicity induced by Enfortumab Vedotin through sequential adaptation and combination with immune checkpoint inhibitors. Cureus 17:e89678. 10.7759/cureus.89678 40926943 10.7759/cureus.89678 PMC12416118

[6] Huang L, Zhang S, Sun L et al (2025) Immune checkpoint inhibitors in Epidermal Growth Factor Receptor-Tyrosine Kinase Inhibitor-resistant chemotherapy-naïve advanced Non-Small Cell Lung Cancer: a meta-analysis based on eight randomized trials. JCO Precis Oncol 9:e2400907. 10.1200/PO-24-00907 40638873 10.1200/PO-24-00907 PMC12262128

[7] Hellmann MD, Paz-Ares L, Bernabe Caro R et al (2019) Nivolumab plus Ipilimumab in Advanced Non-Small-Cell Lung Cancer. N Engl J Med 381:2020–2031. 10.1056/NEJMoa1910231 31562796 10.1056/NEJMoa1910231

[8] Paz-Ares L, Ciuleanu TE, Cobo M et al (2021) First-line nivolumab plus ipilimumab combined with two cycles of chemotherapy in patients with non-small-cell lung cancer (CheckMate 9LA): an international, randomised, open-label, phase 3 trial. Lancet Oncol 22:198–211. 10.1016/S1470-2045(20)30641-0 33476593 10.1016/S1470-2045(20)30641-0

[9] Viscuse PV, Marques-Piubelli ML, Heberton MM et al (2021) Case report: Enfortumab Vedotin for metastatic urothelial carcinoma: a case series on the clinical and histopathologic spectrum of adverse cutaneous reactions from fatal Stevens-Johnson Syndrome/Toxic Epidermal Necrolysis to dermal hypersensitivity reaction. Front Oncol 11:621591. 10.3389/fonc.2021.621591 33747934 10.3389/fonc.2021.621591 PMC7970171

[10] Brower B, McCoy A, Ahmad H et al (2024) Managing potential adverse events during treatment with enfortumab vedotin + pembrolizumab in patients with advanced urothelial cancer. Front Oncol 14:1326715. 10.3389/fonc.2024.1326715 38711854 10.3389/fonc.2024.1326715 PMC11071165

[11] Iimura Y, Iihara H, Saito Y, Nomura H, Iwamoto T, Kotera M, Tsuchiya Y, Sumiya T, Kono M, Hirate D, Kurokawa T, Hayashi T, Hashimoto H, Higuchi J, Urakawa R, Saotome H, Kuroda S; Japanese Pharmacist-led Oncodermatology Study Team (J-PLOS) (2025) Chemotherapy-related hand-foot syndrome and hand-foot skin reaction: a review of management and possible approaches for Asian patients by the Japanese pharmacist-led oncodermatology study team. Int J Clin Oncol 30:2474–2488. 10.1007/s10147-025-02895-y 41053386 10.1007/s10147-025-02895-y PMC12644181

[12] Iimura Y, Iihara H, Saito Y et al (2025) Epidermal growth factor receptor inhibitor-related skin toxicities: a review of management and possible preventive and therapeutic approaches for Asian patients by the Japanese Pharmacist-led Oncodermatology Study Team. Int J Clin Oncol 30:2192–2207. 10.1007/s10147-025-02868-1 40888993 10.1007/s10147-025-02868-1 PMC12568918

[13] LE Wiznia EN (2017) Differences in skin structure and function in ethnic populations. In: Vashi NA, Maibach HI (eds) Dermatoanthropology of ethnic skin and hair. Springer International Publishing, Cham, pp 35–48

[14] Taylor SC (2002) Skin of color: biology, structure, function, and implications for dermatologic disease. J Am Acad Dermatol 46:S41–S62. 10.1067/mjd.2002.120790 11807469 10.1067/mjd.2002.120790

[15] Adegbenro A, Taylor S (2013) Structural, physiological, functional, and cultural differences in skin of color. In: Alexis AF, Barbosa VH (eds) Skin of color: a practical guide to dermatologic diagnosis and treatment. Springer New York, New York, NY, pp 1–19

[16] Goldinger SM, Stieger P, Meier B et al (2016) Cytotoxic cutaneous adverse drug reactions during Anti-PD-1 therapy. Clin Cancer Res 22:4023–4029. 10.1158/1078-0432.CCR-15-2872 26957557 10.1158/1078-0432.CCR-15-2872

[17] Schaberg KB, Novoa RA, Wakelee HA et al (2016) Immunohistochemical analysis of lichenoid reactions in patients treated with anti-PD-L1 and anti-PD-1 therapy. J Cutan Pathol 43:339–346. 10.1111/cup.12666 26762844 10.1111/cup.12666

[18] Minkis K, Garden BC, Wu S et al (2013) The risk of rash associated with ipilimumab in patients with cancer: a systematic review of the literature and meta-analysis. J Am Acad Dermatol 69:e121–e128. 10.1016/j.jaad.2012.12.963 23357570 10.1016/j.jaad.2012.12.963

[19] Lacouture ME, Wolchok JD, Yosipovitch G et al (2014) Ipilimumab in patients with cancer and the management of dermatologic adverse events. J Am Acad Dermatol 71:161–169. 10.1016/j.jaad.2014.02.035 24767731 10.1016/j.jaad.2014.02.035

[20] Perret RE, Josselin N, Knol AC et al (2017) Histopathological aspects of cutaneous erythematous-papular eruptions induced by immune checkpoint inhibitors for the treatment of metastatic melanoma. Int J Dermatol 56:527–533. 10.1111/ijd.13540 28188628 10.1111/ijd.13540

[21] Joseph RW, Cappel M, Goedjen B et al (2015) Lichenoid dermatitis in three patients with metastatic melanoma treated with anti-PD-1 therapy. Cancer Immunol Res 3:18–22. 10.1158/2326-6066.CIR-14-0134 25287118 10.1158/2326-6066.CIR-14-0134

[22] Shi VJ, Rodic N, Gettinger S et al (2016) Clinical and histologic features of lichenoid mucocutaneous eruptions due to Anti-Programmed Cell Death 1 and Anti-Programmed Cell Death Ligand 1 immunotherapy. JAMA Dermatol 152:1128–1136. 10.1001/jamadermatol.2016.2226 27411054 10.1001/jamadermatol.2016.2226 PMC6108080

[23] Weber JS, Hodi FS, Wolchok JD et al (2017) Safety profile of nivolumab monotherapy: A pooled analysis of patients with advanced melanoma. J Clin Oncol 35:785–792. 10.1200/JCO.2015.66.1389 28068177 10.1200/JCO.2015.66.1389

[24] Weber JS, Kähler KC, Hauschild A (2012) Management of immune-related adverse events and kinetics of response with Ipilimumab. J Clin Oncol 30:2691–2697. 10.1200/JCO.2012.41.6750 22614989 10.1200/JCO.2012.41.6750

[25] Hassel JC, Heinzerling L, Aberle J et al (2017) Combined immune checkpoint blockade (anti-PD-1/anti-CTLA-4): evaluation and management of adverse drug reactions. Cancer Treat Rev 57:36–49. 10.1016/j.ctrv.2017.05.003 28550712 10.1016/j.ctrv.2017.05.003

[26] Freeman-Keller M, Kim Y, Cronin H et al (2016) Nivolumab in resected and unresectable metastatic melanoma: characteristics of immune-related adverse events and association with outcomes. Clin Cancer Res 22:886–894. 10.1158/1078-0432.CCR-15-1136 26446948 10.1158/1078-0432.CCR-15-1136 PMC4755809

[27] Belum VR, Benhuri B, Postow MA et al (2016) Characterisation and management of dermatologic adverse events to agents targeting the PD-1 receptor. Eur J Cancer 60:12–25. 10.1016/j.ejca.2016.02.010 27043866 10.1016/j.ejca.2016.02.010 PMC4998047

[28] Sanlorenzo M, Vujic I, Daud A et al (2015) Pembrolizumab cutaneous adverse events and their association with disease progression. JAMA Dermatol 151:1206–1212. 10.1001/jamadermatol.2015.1916 26222619 10.1001/jamadermatol.2015.1916 PMC5061067

[29] Van Doorn R, Kirtschig G, Scheffer E et al (2002) Follicular and epidermal alterations in patients treated with ZD1839 (Iressa), an inhibitor of the epidermal growth factor receptor. Br J Dermatol 147:598–601. 10.1046/j.1365-2133.2002.04864.x 12207609 10.1046/j.1365-2133.2002.04864.x

[30] Busam KJ, Capodieci P, Motzer R et al (2001) Cutaneous side-effects in cancer patients treated with the antiepidermal growth factor receptor antibody C225. Br J Dermatol 144:1169–1176. 10.1046/j.1365-2133.2001.04226.x 11422037 10.1046/j.1365-2133.2001.04226.x

[31] Pascual JC, Belinchón I, Sivera F et al (2005) Severe cutaneous toxicity following treatment with Gefitinib (ZD1839). Br J Dermatol 153:1222–1223 16307664 10.1111/j.1365-2133.2005.06885.x

[32] Guy R, Kealey T (1998) Modelling the infundibulum in acne. Dermatology 196:32–37. 10.1159/000017862 9557221 10.1159/000017862

[33] Mascia F, Mariani V, Girolomoni G et al (2003) Blockade of the EGF receptor induces a deranged chemokine expression in keratinocytes leading to enhanced skin inflammation. Am J Pathol 163:303–312. 10.1016/S0002-9440(10)63654-1 12819035 10.1016/S0002-9440(10)63654-1 PMC1868171

[34] Baby B, Sam N, Pn M et al (2025) Therapy-related hand-foot syndrome: a review. J Chemother 37:567–578. 10.1080/1120009X.2024.2437336 39651796 10.1080/1120009X.2024.2437336

[35] Diasio RB (2000) Oral DPD-inhibitory fluoropyrimidine drugs. Oncology (Williston Park) 14:19–23 11098485

[36] Degen A, Alter M, Schenck F et al (2010) The hand-foot-syndrome associated with medical tumor therapy - classification and management. J Dtsch Dermatol Ges 8:652–661. 10.1111/j.1610-0387.2010.07449.x 20482685 10.1111/j.1610-0387.2010.07449.x

[37] Yokomichi N, Nagasawa T, Coler-Reilly A et al (2013) Pathogenesis of hand-foot syndrome induced by PEG-modified liposomal Doxorubicin. Hum Cell 26:8–18. 10.1007/s13577-012-0057-0 23386177 10.1007/s13577-012-0057-0 PMC3595474

[38] Lou Y, Wang Q, Zheng J et al (2016) Possible pathways of Capecitabine-induced hand-foot syndrome. Chem Res Toxicol 29:1591–1601. 10.1021/acs.chemrestox.6b00215 27631426 10.1021/acs.chemrestox.6b00215

[39] Santhosh A, Sharma A, Bakhshi S et al (2024) Topical Diclofenac for prevention of Capecitabine-associated hand-foot syndrome: a double-blind randomized controlled trial. J Clin Oncol 42:1821–1829. 10.1200/JCO.23.01730 38412399 10.1200/JCO.23.01730

[40] Iimura Y, Baba K, Furukawa N et al (2025) A phase II study evaluating the preventive effect of topical Hydrocortisone for Capecitabine-induced hand-foot syndrome in patients with colorectal cancer receiving adjuvant chemotherapy with Capecitabine plus Oxaliplatin (T-CRACC study). Int J Clin Oncol 30:2316–2324. 10.1007/s10147-025-02857-4 40810825 10.1007/s10147-025-02857-4 PMC12568862

[41] Iimura Y, Shimomura H, Yasu T et al (2018) NSAIDs may prevent EGFR-TKI-related skin rash in non-small cell lung cancer patients. Int J Clin Pharmacol Ther 56:551–554. 10.5414/CP203323 30232957 10.5414/CP203323

[42] Tanaka R, Ishikawa H, Sato J et al (2022) Prevention of acne-like eruption caused by Panitumumab treatment through oral administration of non-steroidal anti-inflammatory drugs. Biol Pharm Bull 45:1531–1536. 10.1248/bpb.b22-00404 36184512 10.1248/bpb.b22-00404

[43] McLellan B, Ciardiello F, Lacouture ME et al (2015) Regorafenib-associated hand-foot skin reaction: practical advice on diagnosis, prevention, and management. Ann Oncol 26:2017–2026. 10.1093/annonc/mdv244 26034039 10.1093/annonc/mdv244 PMC4576906

[44] Belum VR, Wu S, Lacouture ME (2013) Risk of hand-foot skin reaction with the novel multikinase inhibitor Regorafenib: a meta-analysis. Invest New Drugs 31:1078–1086. 10.1007/s10637-013-9977-0 23700287 10.1007/s10637-013-9977-0

[45] Gaumond SI, Beraja GE, Kamholtz I et al (2025) Chemotherapy-induced alopecia in ovarian cancer: incidence, mechanisms, and impact across treatment regimens. Cancers (Basel) 17:411. 10.3390/cancers17030411 39941780 10.3390/cancers17030411 PMC11816305

[46] Perez AM, Haberland NI, Miteva M et al (2024) Chemotherapy-induced alopecia by Docetaxel: prevalence, treatment and prevention. Curr Oncol 31:5709–5721. 10.3390/curroncol31090423 39330051 10.3390/curroncol31090423 PMC11431623

[47] Hirotsu KE, Rana J, Wang JY et al (2021) Clinicopathologic characterization of Enfortumab Vedotin-associated cutaneous toxicity in patients with urothelial carcinoma. J Am Acad Dermatol 85:1610–1611. 10.1016/j.jaad.2020.11.067 33301805 10.1016/j.jaad.2020.11.067

[48] Rosenberg J, Sridhar SS, Zhang J et al (2020) EV-101: a Phase I study of single-agent Enfortumab Vedotin in patients with Nectin-4-Positive solid tumors, including metastatic urothelial carcinoma. J Clin Oncol 38:1041–1049. 10.1200/JCO.19.02044 32031899 10.1200/JCO.19.02044 PMC7106979

[49] Egbeto IA, Vlachou E, Herrera DB et al (2024) Clinical and histopathological characterization of Enfortumab Vedotin-associated cutaneous toxicities: a case series. JAAD Case Rep 57:114–121. 10.1016/j.jdcr.2024.12.019 40124881 10.1016/j.jdcr.2024.12.019 PMC11928977

[50] Yang H, Yu X, An Z (2022) Cutaneous toxicity associated with Enfortumab Vedotin: a real-word study leveraging US food and drug administration adverse event reporting system. Front Oncol 11:801199. 10.3389/fonc.2021.801199 35127510 10.3389/fonc.2021.801199 PMC8807512

[51] Shitara K, Rha SY, Wyrwicz L et al (2025) Pembrolizumab plus chemotherapy versus chemotherapy as perioperative therapy in locally advanced gastric and gastroesophageal junction cancer: final analysis of the randomized, Phase III KEYNOTE-585 study. J Clin Oncol 43:3152–3159. 10.1200/JCO-25-00486 40829093 10.1200/JCO-25-00486

[52] Yasui H, Aizawa M, Yamaguchi K et al (2025) First-line Pembrolizumab plus chemotherapy for participants in Japan with gastric or gastroesophageal junction adenocarcinoma: subgroup analysis of the Phase 3 KEYNOTE-859 study. Int J Clin Oncol 30:2003–2011. 10.1007/s10147-025-02847-6 40750941 10.1007/s10147-025-02847-6

[53] Kang YK, Terashima M, Kim YW et al (2024) Adjuvant Nivolumab plus chemotherapy versus placebo plus chemotherapy for Stage III gastric or gastro-oesophageal junction cancer after gastrectomy with D2 or more extensive lymph-node dissection (ATTRACTION-5): a randomised, multicentre, double-blind, placebo-controlled, Phase 3 trial. Lancet Gastroenterol Hepatol 9:705–717. 10.1016/S2468-1253(24)00156-0 38906161 10.1016/S2468-1253(24)00156-0

[54] Satake H, Lee KW, Chung HC et al (2023) Pembrolizumab or Pembrolizumab plus chemotherapy versus standard of care chemotherapy in patients with advanced gastric or gastroesophageal junction adenocarcinoma: Asian subgroup analysis of KEYNOTE-062. Jpn J Clin Oncol 53:221–229. 10.1093/jjco/hyac188 36533429 10.1093/jjco/hyac188 PMC9991501

[55] Liu T, Bai Y, Lin X et al (2023) First-line Nivolumab plus chemotherapy vs chemotherapy in patients with advanced gastric, gastroesophageal junction and esophageal adenocarcinoma: CheckMate 649 Chinese subgroup analysis. Int J Cancer 152:749–760. 10.1002/ijc.34296 36121651 10.1002/ijc.34296 PMC10092493

[56] Kang YK, Ryu MH, Oh DY et al (2025) Nivolumab plus chemotherapy versus placebo plus chemotherapy in Korean patients with HER2-Negative, untreated, unresectable advanced or recurrent gastric or gastroesophageal junction cancer: subgroup analysis of a randomized, multicenter, double-blind Phase 3 trial. Lancet Oncol 23(2):234–247. 10.1016/S1470-2045(21)00692-6 10.1016/S1470-2045(21)00692-6 35030335

[57] Shitara K, Van Cutsem E, Bang YJ et al (2020) Efficacy and safety of pembrolizumab or pembrolizumab plus chemotherapy vs chemotherapy alone for patients with first-line, advanced gastric cancer: The KEYNOTE-062 phase 3 randomized clinical trial. JAMA Oncol 6:1571–1580. 10.1001/jamaoncol.2020.3370 32880601 10.1001/jamaoncol.2020.3370 PMC7489405

[58] Janjigian YY, Shitara K, Moehler M et al (2021) First-line nivolumab plus chemotherapy versus chemotherapy alone for advanced gastric, gastro-oesophageal junction, and oesophageal adenocarcinoma (CheckMate 649): A randomised, open-label, phase 3 trial. Lancet 398:27–40. 10.1016/S0140-6736(21)00797-2 34102137 10.1016/S0140-6736(21)00797-2 PMC8436782

[59] Janjigian YY, Kawazoe A, Bai Y et al (2023) Pembrolizumab plus trastuzumab and chemotherapy for HER2-positive gastric or gastro-oesophageal junction adenocarcinoma: interim analyses from the phase 3 KEYNOTE-811 randomised placebo-controlled trial. Lancet 402:2197–2208. 10.1016/S0140-6736(23)02033-0 37871604 10.1016/S0140-6736(23)02033-0

[60] Qiu MZ, Oh DY, Kato K et al (2024) Tislelizumab plus chemotherapy versus placebo plus chemotherapy as first line treatment for advanced gastric or gastro-oesophageal junction adenocarcinoma: RATIONALE-305 randomised, double blind, phase 3 trial. BMJ 385:e078876. 10.1136/bmj-2023-078876 38806195 10.1136/bmj-2023-078876

[61] Llovet JM, Kudo M, Merle P et al (2023) Lenvatinib plus pembrolizumab versus lenvatinib plus placebo for advanced hepatocellular carcinoma (LEAP-002): a randomised, double-blind, phase 3 trial. Lancet Oncol 24:1399–1410. 10.1016/S1470-2045(23)00469-2 38039993 10.1016/S1470-2045(23)00469-2

[62] Pal SK, Albiges L, Tomczak P et al (2023) Atezolizumab plus cabozantinib versus cabozantinib monotherapy for patients with renal cell carcinoma after progression with previous immune checkpoint inhibitor treatment (CONTACT-03): A multicentre, randomised, open-label, phase 3 trial. Lancet 402:185–195. 10.1016/S0140-6736(23)00922-4 37290461 10.1016/S0140-6736(23)00922-4 PMC11017728

[63] Nishio S, Yonemori K, Usami T et al (2022) Pembrolizumab plus chemotherapy in Japanese patients with persistent, recurrent or metastatic cervical cancer: Results from KEYNOTE-826. Cancer Sci 113:3877–3887. 10.1111/cas.15479 35792064 10.1111/cas.15479 PMC9633308

[64] Saji S, Ohsumi S, Ito M et al (2022) Subgroup analysis of Japanese patients in a phase III randomized, controlled study of neoadjuvant atezolizumab or placebo, combined with nab-paclitaxel and anthracycline-based chemotherapy in early triple-negative breast cancer (IMpassion031). Jpn J Clin Oncol 52:1124–1133. 10.1093/jjco/hyac098 35750038 10.1093/jjco/hyac098 PMC9538755

[65] Emens LA, Adams S, Barrios CH et al (2021) First-line atezolizumab plus nab-paclitaxel for unresectable, locally advanced, or metastatic triple-negative breast cancer: IMpassion130 final overall survival analysis. Ann Oncol 32:983–993. 10.1016/j.annonc.2021.05.355 34272041 10.1016/j.annonc.2021.05.355

[66] Petrylak DP, Ratta R, Matsubara N et al (2025) Pembrolizumab plus docetaxel versus docetaxel for previously treated metastatic castration-resistant prostate cancer: The randomized, double-blind, phase III KEYNOTE-921 trial. J Clin Oncol 43:1638–1649. 10.1200/JCO-24-01283 40043230 10.1200/JCO-24-01283 PMC12058370

[67] Schmid P, Rugo HS, Adams S et al (2020) Atezolizumab plus nab-paclitaxel as first-line treatment for unresectable, locally advanced or metastatic triple-negative breast cancer (IMpassion130): updated efficacy results from a randomised, double-blind, placebo-controlled, phase 3 trial. Lancet Oncol 21:44–59. 10.1016/S1470-2045(19)30689-8 31786121 10.1016/S1470-2045(19)30689-8

[68] Cortes J, Cescon DW, Rugo HS et al (2020) Pembrolizumab plus chemotherapy versus placebo plus chemotherapy for previously untreated locally recurrent inoperable or metastatic triple-negative breast cancer (KEYNOTE-355): a randomised, placebo-controlled, double-blind, phase 3 clinical trial. Lancet 396:1817–1828. 10.1016/S0140-6736(20)32531-9 33278935 10.1016/S0140-6736(20)32531-9

[69] West H, McCleod M, Hussein M et al (2019) Atezolizumab in combination with carboplatin plus nab-paclitaxel chemotherapy compared with chemotherapy alone as first-line treatment for metastatic non-squamous non-small-cell lung cancer (IMpower130): A multicentre, randomised, open-label, phase 3 trial. Lancet Oncol 20:924–937. 10.1016/S1470-2045(19)30167-6 31122901 10.1016/S1470-2045(19)30167-6

[70] Govindan R, Szczesna A, Ahn MJ et al (2017) Phase III trial of ipilimumab combined with paclitaxel and carboplatin in advanced squamous non-small-cell lung cancer. J Clin Oncol 35:3449–3457. 10.1200/JCO.2016.71.7629 28854067 10.1200/JCO.2016.71.7629

[71] Sugawara S, Lee JS, Kang JH et al (2021) Nivolumab with carboplatin, paclitaxel, and bevacizumab for first-line treatment of advanced nonsquamous non-small-cell lung cancer. Ann Oncol 32:1137–1147. 10.1016/j.annonc.2021.06.004 34139272 10.1016/j.annonc.2021.06.004

[72] Wang ZX, Cui C, Yao J et al (2022) Toripalimab plus chemotherapy in treatment-naïve, advanced esophageal squamous cell carcinoma (JUPITER-06): A multi-center phase 3 trial. Cancer Cell 40:277-288.e3. 10.1016/j.ccell.2022.02.007 35245446 10.1016/j.ccell.2022.02.007

[73] Wang J, Lu S, Yu X et al (2021) Tislelizumab plus chemotherapy vs chemotherapy alone as first-line treatment for advanced squamous non-small-cell lung cancer: A phase 3 randomized clinical trial. JAMA Oncol 7:709–717. 10.1001/jamaoncol.2021.0366 33792623 10.1001/jamaoncol.2021.0366 PMC8017481

[74] Xu J, Kato K, Raymond E et al (2023) Tislelizumab plus chemotherapy versus placebo plus chemotherapy as first-line treatment for advanced or metastatic oesophageal squamous cell carcinoma (RATIONALE-306): A global, randomised, placebo-controlled, phase 3 study. Lancet Oncol 24:483–495. 10.1016/S1470-2045(23)00108-0 37080222 10.1016/S1470-2045(23)00108-0

[75] Yang JC, Shepherd FA, Kim DW et al (2019) Osimertinib plus durvalumab versus osimertinib monotherapy in EGFR T790M-positive NSCLC following previous EGFR TKI therapy: CAURAL brief report. J Thorac Oncol 14:933–939. 10.1016/j.jtho.2019.02.001 30763730 10.1016/j.jtho.2019.02.001

